# Mixing Exploitation and Conservation: A Recipe for Disaster

**DOI:** 10.1371/journal.pbio.0040418

**Published:** 2006-11-14

**Authors:** Liza Gross

Most governments around the world set conservation policy based on the assumption that resource exploitation and species protection can co-exist in the same place. These policies have led to Orwellian “marine protected areas” that host commercial fishing operations, leading one to wonder who’s protecting whom. A new study reveals the danger of this approach and shows that it’s time to let protection mean protection.

For decades, the Dutch government sanctioned mechanical cockle dredging in three-fourths of the intertidal flats of the Wadden Sea—a natural monument protected under two intergovernmental treaties. Before suction dredging began in the 1960s, an estimated 2,000 tons of cockles were hand-harvested from the reserve each year. In 1989, the high-pressure, motor-driven water pumps used in suction dredging sucked up close to 80,000 tons of cockles. By 2004, the Dutch government decided the environmental costs were too great and stopped the practice.

Jan van Gils and colleagues investigated the ecological impacts of commercial cockle dredging on intertidal ecosystems by studying a long-distance migrant shorebird that dines principally on cockles, the red knot *(Calidris canutus islandica)*. Up to 50% of the global red knot population uses the Dutch Wadden Sea at some point during their annual cycle.

Red knots are exquisitely adapted to their lifestyle. They have a pressure-sensitive bill that senses hard objects buried in the sand and a shell-crushing gizzard to accommodate the birds’ penchant for swallowing their catch whole. They even have a flexible digestive system that minimizes the energy costs of flying up to 16,000 kilometers between their arctic breeding grounds and winter homes in Europe and the tropics—their gizzard expands and contracts to balance daily food intake and energy needs.

To determine the effects of dredging on the birds, the authors sampled prey quality and density over 2,800 Wadden Sea sites during the late summer months (late July to early September) for five years starting in 1998. Dredging occurred each year from September to December, immediately after their sample collections. In undredged areas, cockle densities increased by 2.6% each year, and the quality remained stable. In dredged areas, cockle densities remained stable, and their quality (flesh-to-shell ratio) declined by 11.3% each year—paralleling the decline in the quality of the birds’ diet (as measured by droppings). This finding falls in line with evidence that dredging disturbs the silt cockles like to settle in, as well as their feeding conditions—which in turn reduces their quality as a food resource.

Based on prey quality and densities, Van Gils et al. predicted the energy intake rate for knots with an average-size gizzard at each site (all sites were pooled into 272 blocks, each with an area of 1 square kilometer), then calculated the percentage of blocks that would not yield sufficient intake rates for knots to avoid starvation. From 1998 to 2002, the percentage of blocks that couldn’t sustain knots increased from 66% to 87%—all attributable to dredging in previously suitable sites. Reduced prey density caused some of this degradation, but most stemmed from declines in both cockle density and quality.

**Figure:**
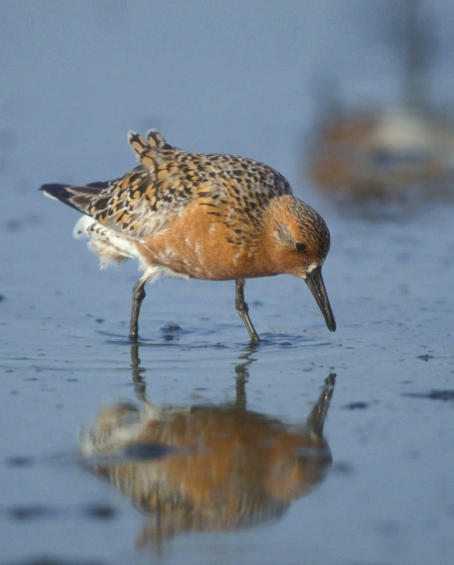
Commercial shellfish dredging in the Dutch Wadden Sea led to declines in both the quality and amount of the red knot's food resources, causing the population to crash. (Photo: Jan van de Kam)

The authors caught and color-banded the birds so they could estimate survival rates the following year, and they measured gizzard mass with ultrasonography. As expected, when prey quality declined, birds needed larger gizzards to process the relatively higher proportion of shells in their diet. Their chances of surviving conditions at the Wadden Sea increased as a function of prey quality and gizzard flexibility. Birds that did not return had much smaller gizzards than those that did. Survival rate calculations based on gizzard size and prey quality revealed that if birds could not expand their gizzard and prey quality was low (0.15 grams of flesh per gram of shell), only 47% of arriving birds would avoid starvation. A much greater proportion would survive if their gizzard could expand by at least 1 gram (70% for 1 gram, 88% for 2 grams).

These degraded food conditions, the authors conclude, explains why red knot populations have declined by 80% in the Wadden Sea. And increased mortality in the Wadden Sea—which the authors estimate at 58,000 birds over five years—accounts for the 25% decline of red knots across their entire northwest European wintering grounds. Dredging reduced the quality of red knots’ primary food source so drastically that even the birds’ extraordinarily adaptable digestive system could not save them. The authors point out that dredging doesn’t even provide significant economic benefits—only 11 outfits manage 22 fishing boats—yet is “directly responsible” for the widespread decline of a protected shorebird. These findings put the lie to the notion that commercial exploitation is consistent with conservation and underscore the risks of disturbing critical habitat for threatened or endangered species.

